# In Silico Digital Breast Tomosynthesis Dataset for the Comparative Analysis of Deep Learning Models in Tumor Segmentation

**DOI:** 10.1007/s10278-025-01626-z

**Published:** 2025-08-04

**Authors:** Cristina Alfaro Vergara, Nicolás Araya Caro, Domingo Mery Quiroz, Claudia Prieto Vasquez

**Affiliations:** 1 Department of Medical Technology, Faculty of Health Sciences, Universidad de Tarapacá, Arica, Chile; 2 Institute for Biological and Medical Engineering, Pontificia Universidad Católica de Chile, Santiago, Chile; 3 Department of Computer Sciences, Faculty of Engineering, Pontificia Universidad Católica de Chile, Santiago, Chile; 4 Millennium Institute for Intelligent Healthcare Engineering i-Health, Santiago, Chile; 5 School of Engineering, Pontificia Universidad Católica de Chile, Santiago, Chile

**Keywords:** Breast tumor segmentation, Digital breast tomosynthesis (DBT), In silico data, Deep learning U-Net, Hybrid training

## Abstract

The scarcity of publicly available digital breast tomosynthesis (DBT) datasets significantly limits the development of robust deep learning (DL) models for breast tumor segmentation. In this exploratory proof-of-concept study, we assess the viability of in silico-generated DBT data as a training source for tumor segmentation. A dataset of 230 two-dimensional (2D) regions of interest (ROIs) derived from FDA-cleared software and encompassing a spectrum of breast densities and tumor complexities, was used to train 13 DL models, including U-Net, FCN, DeepLabv3, and DeepLabv3 + architectures. Each model was trained either from scratch or fine-tuned using COCO-pretrained weights (ResNet50/101 backbones). Performance was evaluated using F1-score, intersection over union (IoU), precision, and recall. Among all models, U-Net trained from scratch and DeepLabv3 + fine-tuned with ResNet50 achieved the highest and most consistent results (F1-scores of 82.52% and 84.98%, and per-image IoUs of 78.49% and 83.77%, respectively). No statistically significant differences were found using the Wilcoxon signed-rank test and post hoc Bonferroni correction (*α* > 0.0042). To evaluate generalization across domains, the baseline U-Net model was retrained from scratch on a hybrid dataset combining in silico and real-world DBT ROIs, yielding promising results (F1-score of 79%). Despite the domain shift, these findings support the utility of in silico DBT as a complementary resource for training and benchmarking DL models, particularly in data-limited environments. This study provides foundational experimental evidence for integrating computationally generated in silico data into AI-based DBT tumor segmentation research workflows.

## Introduction

Breast cancer remains the leading cause of cancer-related mortality among women worldwide [1]. Thus, early detection and diagnosis are essential to improve prognosis and avoid radical mastectomies, lymphadenectomy, and metastasis. Digital two-dimensional (2D) mammography (DM) has been the reference standard of breast cancer screening for decades, offering a reliable method for detecting abnormalities within breast tissue [2]. However, DM has important limitations due to tissue overlapping, making detection of suspicious lesions difficult, especially in patients with dense breast tissue.

Over the past few years, Digital Breast Tomosynthesis (DBT) has emerged as a complementary modality to DM. This modality provides pseudo-three-dimensional (3D) imaging that improves lesion visibility and reduces the effect of overlapping tissue [3]. Moreover, several prospective and retrospective multicenter screening trials have shown that combining DBT with DM results in significant gains in sensitivity and specificity, especially in patients with dense breasts [4, 5].

However, while DBT offers significant advantages, such as improved tumor margin characterization, it also presents limitations, such as significantly longer reading times, leading to a more labor-intensive diagnostic workload for radiologists. In this context, automating diagnostic tasks such as detection, classification, and segmentation is paramount to supporting clinicians and reducing workload [6].

Furthermore, it is crucial to mention that accurate breast tumor segmentation is a critical task in medical imaging, as it enables precise delineation of tumor boundaries, which is essential for effective diagnosis, treatment planning, and monitoring of breast cancer. By accurately identifying size, shape, and tumor location, clinicians can make informed decisions regarding surgical interventions, radiotherapy, and chemotherapy, ultimately improving patient outcomes [7]. Moreover, precise segmentation facilitates the assessment of tumor response to treatment and aids in the early detection of recurrences [8].

In recent years, deep learning algorithms have shown promise in automating detection, classification, and segmentation processes in breast cancer imaging [9]. However, several remaining challenges must be addressed to transfer its clinical application into the daily clinical routine [10]. The requirement for large datasets is essential for training deep learning models to achieve robust performance. The scarcity and low availability of high-quality annotated and interoperable datasets are particularly notorious in breast imaging [11]. This fact is mainly due to patient privacy concerns, regulatory barriers, and the labor-intensive nature of manual expert annotation [12]. Indeed, only a few public DBT datasets are currently available but often present reduced access to the actual percentage of diverse training examples [13]. On the other hand, other DBT datasets might be skewed to a specific population [14], making their use difficult to generalize to other ethnically diverse or underrepresented populations. In addition, one main constraint is data privacy, either due to restricted public access or due to data sharing regulations, making the reproducibility of those studies challenging [15]. Another major barrier is that the annotation of large-scale datasets relies on the expert yet subjective human perception, making the segmentation reference standard prone to inter- and intra-observer variability [16].

Traditionally, an alternative method to tackle the scarcity of medical imaging datasets has consisted of leveraging transfer learning techniques via fine-tuning pretrained deep learning architectures on large-scale datasets from natural images such as ImageNet [17] or common object in context (COCO) [18]. However, transfer learning from pretrained models has been controversial. While some studies have reported its great potential and effectiveness in classification and detection tasks [19], other works have revealed this method to be ineffective, arguing that this is due to the mismatch in learned features between natural and medical images and therefore, suffering inherently from the domain shift problem [20].

An emerging and rapidly growing field of interest within the medical imaging community has been the use of synthetic data. This approach is currently gaining popularity across various research domains; however, inconsistent terminology has hindered a unified and systematic approach to its study in radiology [21]. As the artificial intelligence (AI) medical imaging community has focused on generative models for synthetic data creation through Generative Adversarial Networks (GANs) and their variants [22, 23], in contrast, the biomedical and clinical fields have emphasized digital twins [24]. While in the specific field of breast imaging, prior studies have advanced in applying GANs to address concerns such as data scarcity via data augmentation or data sharing [25], several drawbacks regarding this type of architecture are its inherent instability in the training process and the non-convergence problem [26]. On the other hand, diffusion models have emerged as a promising approach in image segmentation research by synthesizing the labeled data and obviating the necessity for pixel-annotated data. However, its limitations include a slower generation process relative to other generative models, lower likelihood estimation capabilities, and the inability to perform dimensionality reduction [27].

Recently, a methodology called Virtual Clinical Trials (VCTs) (also known as in- silico imaging trials or virtual imaging trials) is rapidly gaining popularity across multiple medical imaging modalities [28], as it enables the reduction of cost, ethical restrictions, and time requirements among other main constraints encountered when performing real-life medical imaging clinical trials. Particularly in breast imaging, VCT tools development is rapidly advancing [29], offering a potential solution to create an alternative data source. These computational models aim to simulate the anatomical and pathological characteristics of the human breast, potentially providing an abundant and controllable source of imaging simulations in a safer, scalable, and cost-saving way [30].

While primarily VCTs have been designed for the technical evaluation and validation of imaging devices or radiation dose evaluation [31], research focused on leveraging in silico data for AI-based tasks is a very recent and emerging field of study. Although some research groups have worked on building and sharing in silico datasets [31–33] or in developing computational tools to create in silico breast image replicas to advance in the field [31, 34, 35], we identified very few studies aiming to explore in silico data effectiveness on deep learning breast lesions for classification or detection tasks [36, 37] or as a data augmentation strategy for Digital 2D mammography datasets [38]. Moreover, regarding segmentation tasks, we only identified one DBT study using an in silico segmentation approach focused on assessing volumetric breast density [39]. Despite the findings above, the potential of in silico-generated DBT data for training deep learning models specifically aimed at breast tumor segmentation remains largely underexplored. This represents a significant gap in the current literature that warrants further investigation.

Therefore, this study aims to experimentally investigate the reliability of using an in silico-generated DBT dataset for tumor segmentation by assessing the performance of deep learning models trained from scratch and their counterparts pretrained on large-scale natural images and fine-tuned for in silico data. For this purpose, we defined the U-Net architecture [40] as the baseline method to perform the comparative analysis, since it has been considered the most widely adopted and well-established segmentation architecture due to its flexibility, modular design, and effectiveness across various medical imaging modalities [41]. Finally, we evaluated the domain shift generalization capability by assessing the performance of the baseline U-Net architecture using a hybrid approach that integrates both in silico and real-world DBT data. Our findings aim to demonstrate the feasibility of in silico DBT as a complementary training resource for segmentation tasks in data-constrained settings and provide insights into the capabilities and limitations of deep learning models trained on computationally simulated breast images.

## Materials and Methods

**Fig. 1:**
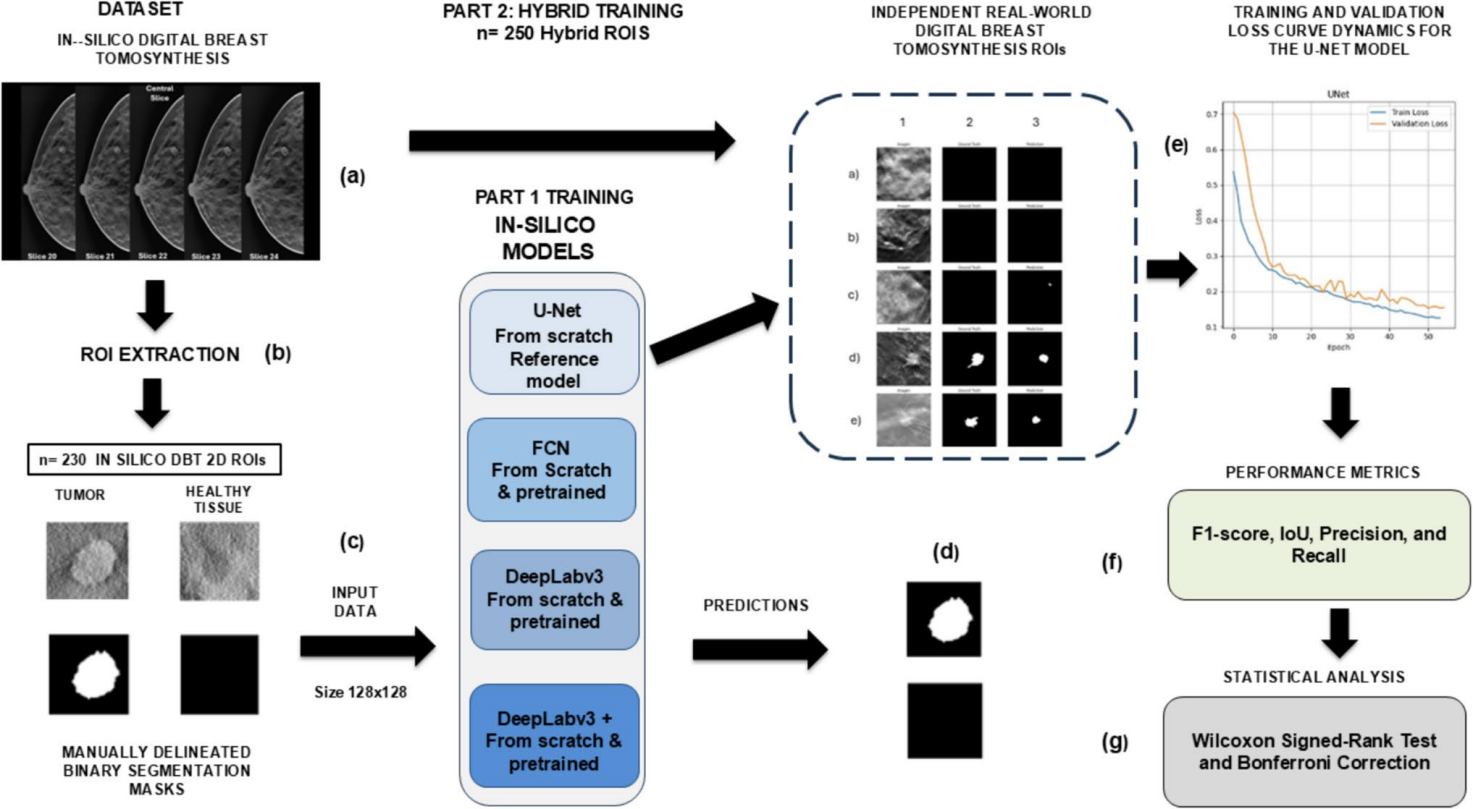
Workflow for our work: The pipeline consists of two parts. (**a**) In silico DBT volumes were used to extract VOIs, which were then decomposed into 2D ROIs. (**b**) A dataset of 230 annotated ROIs was curated, each ROI comprising tumor or healthy tissue. (**c**) These were resized and used as input for model training. (**d**) Four deep learning architectures (U-Net, FCN, DeepLabv3, DeepLabv3 +) were trained from scratch or fine-tuned with ResNet 50/101 as backbones. (**e**) The U-Net model was later retrained using a hybrid dataset (*n* = 250, in silico + real-world DBT ROIs) and tested on unseen independent real data. (**f**) Performance was evaluated using standard metrics (F1-score, IoU, precision, recall), and (g) statistical comparisons were conducted using the Wilcoxon Signed-Rank Test with Bonferroni correction

The first part of this section describes the methodology for the in silico DBT dataset creation, mask delineation, reference standard definition, deep learning architectures selection, training/validation and test procedure, and performance metrics results. Additionally, we describe the statistical analysis to verify statistical significance. Our primary objective was to experimentally evaluate the outcomes of different well-established deep learning architectures when trained either from scratch or pretrained/fine-tuned on a natural images dataset and fed with a full in silico DBT dataset. Thus, we investigated the viability of this method as an in silico experimental proof-of-concept. In the second part, we aimed to assess the performance of a baseline UNet architecture trained from scratch on a hybrid dataset, comprising both in silico and real-world DBT data. Thus, we evaluated the utility of in silico DBT as a complementary resource for training and benchmarking DL models, particularly in data-limited environments. For this purpose, we provide a step-by-step, comprehensive description of our method (Fig. 1), following the recommendations suggested in reference [42].

### In Silico Digital Breast Tomosynthesis Dataset

The breast models, tumors, DBT virtual images, volumes of interest (VOIs), and regions of interest (ROIs) were created by leveraging the VICTRE (Virtual Clinical Trial for Regulatory Evaluation) software [31, 43]. The advantages of the software are as follows:

1. The software is open-source and FDA-cleared.
2. The software comprises several in silico tools enabling running and simulating a full pipeline starting from breast phantom creation, breast compression, lesion creation and insertion, and image generation. It also allows for mimicking real-world DBT device acquisition parameters.
3. Prior studies on similar data derived from the software have demonstrated that the simulated images are sufficiently realistic when compared to real-world datasets, as the images’ properties such as gray levels, contrast-to-noise ratio (CNR), first five statistical moments (mean, variance, skewness, kurtosis, and hyperskewness) and radiomics textures have been assessed, thus offering a very close domain-specific distribution to real-world DBTs [33, 38, 44, 45]. More details on the software and documentation can be found in references [31, 34, 46].

### Breast Phantoms and In Silico DBT

We simulated breast phantoms with diverse glandularity and categorized each phantom based on its fat fraction content. This classification was largely inspired by the four breast parenchymal density categories outlined in the American College of Radiology (ACR) BI-RADS lexicon (Fifth Edition) [47]: fatty, scattered, heterogeneously dense, and dense. While the current BI-RADS lexicon defines these categories as subjective estimates of breast density associated with variations in mammography sensitivity [48], our approach utilized a quantitative descriptor based on the fat fraction simulated for each mathematical phantom. This method aligns closely with the quantitative definitions used in the earlier ACR BI-RADS Fourth Edition lexicon [49]. We simulated a total of 30 breast phantoms, evenly distributed across the four density categories. The breast phantoms have a 0.05 mm^3^ voxel size. The simulated fat fraction ranged from 0.75 to 0.95 for the fatty breast category, 0.55 to 0.70 for scattered, 0.30 to 0.50 for heterogeneously dense, and 0.05 to 0.25 for the dense breast category.

In the next step, we simulated the breast compression thickness between 40 and 65 mm, reflecting the range typically observed in clinical practice [50].

The final step consisted of obtaining the DBT reconstructions derived from the breast phantoms. The VICTRE system employs a 3D filtered back projection (FBP) reconstruction algorithm to generate DBT volumes. The FBP code takes parameters such as system geometry, breast model dimensions, and voxel size. Thus, the reconstructed DBT cases have different dimensions depending on breast density and compression thickness. As an example, a DBT case might have dimensions in the order of magnitude of 1300 pixels (width), 450 pixels (height), and depths ranging from 38 to 68 slices, depending on the simulated breast compression. A more detailed DBT case example is provided in Appendix Fig. 8.

**Fig. 2:**
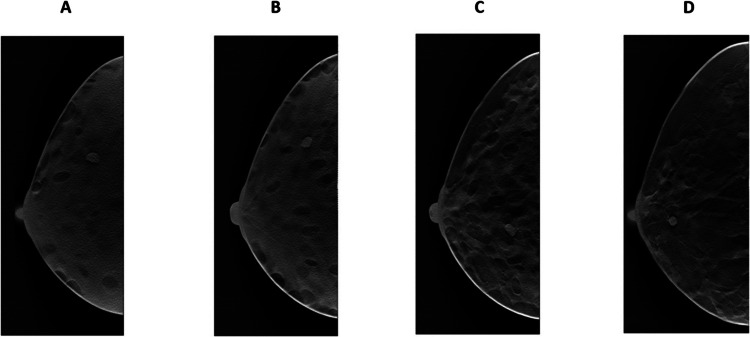
In silico DBT cases reconstructed with the full pipeline of the VICTRE software. **A** to **D** Show examples of the central slice of four different DBT cases across different breast tissue ACR type categories: **A** dense breast tissue, **B** heterogeneously dense breast tissue, **C** scattered breast tissue, and **D** fatty breast tissue. All DBTs shown contain an embedded tumor

As a final step, 30 DBT cases were derived from the 30 simulated breast phantoms. Figure 2 shows the central slices of four in silico DBT cases derived from different phantoms of different tissue density categories.

### Breast Tumors Simulation

We simulated 30 spiculated and irregular-shaped tumors with several densities, mimicking the characteristics described in the literature to be more likely indicators of malignancy, and a density ratio of 1.1, 1.3, and 1.5 relative to that of healthy surrounding glandular tissue. High-density ratio tumors have been described to be more likely malignant [51]. The mean tumor radius ranged from 0.5 mm to 4 mm. We then embedded the tumors into the breast phantoms, resulting in the DBTs illustrated in Fig. 2.

### Volumes of Interest (VOIs) and Regions of Interest (ROIs) Extraction

**Fig. 3:**
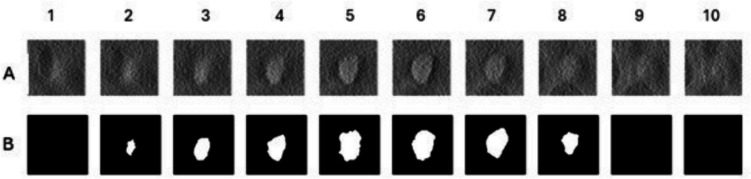
Example of a single volume of interest (VOI) extracted from a DBT volume, containing a 2 mm mean radius tumor. **A** The 109 × 109 × 10 VOI is decomposed into ten 2D regions of interest (ROIs) with a size of 109 × 109 pixels, each treated as an independent input for training. **B** Corresponding binary segmentation masks. Note that each ROI captures different anatomical contexts—central, peripheral, or healthy tissue, the latter represented by an empty mask (columns 1, 9, and 10). This ROI-based method is intended to improve model generalization through diverse learning examples. Column 6 corresponds to the central tumor slice

Due to the high dimensionality of DBT volumes described in previous sections and to ensure anatomical diversity and maximize learning generalization, each 3D volume of interest (VOI) extracted from a simulated DBT volume (size, 109 × 109 × 10) was decomposed into ten 2D regions of interest (ROIs). These ROIs span a spectrum of anatomical contexts, including central tumor slices, peripheral margins, and tumor-free regions composed entirely of healthy tissue. ROIs containing either partial tumor structures or no tumor at all were retained to preserve contextual variability. As illustrated in Fig. 3 (columns 1, 9, and 10), several ROIs represent healthy tissue and are associated with empty binary masks.

Although our training strategy is based on 2D ROIs, these were extracted from full-volume DBT simulations to preserve anatomical realism and intra-volume heterogeneity. Simulating the entire breast volume—rather than isolated patches—enables more realistic modeling of background parenchymal patterns, tumor-to-tissue contrast, and acquisition-related variations such as compression and slice thickness. This approach aligns with VICTRE and other virtual clinical trial frameworks designed to replicate real-world imaging conditions [31, 43]. In the context of data-limited or low-resource environments, the use of 2D ROI-based training from high-fidelity 3D simulations provides a computationally efficient compromise, enabling robust generalization while maintaining close resemblance to clinical data.

Each ROI was treated as an independent training instance to expose the model to both lesion-positive and lesion-negative patterns. This strategy aligns with prior research in breast imaging, showing that parenchymal characteristics from different areas of the breast may contribute differently towards the risk for developing breast cancer [52]. Consequently, patch-based and ROI-wise training approaches leverage local tissue heterogeneity. Moreover, by incorporating a range of radiological contexts, this sampling design supports the development of more robust segmentation models that generalize beyond lesion-centered slices.

### Mask Delineation and Reference Standard Definition

The ROIs were manually segmented by an expert operator using the Image J software [53], where 0 is defined as the background pixels belonging to the healthy surrounding breast tissue, and the foreground is defined as 1 for the pixels belonging to the tumor. Thus, each binary mask was defined as the “Reference Standard,” commonly known as the Ground Truth. Note that, unlike real-world annotations, as the software enables the creation of the ground truth tumor, the operator knows the exact tumor’s location and shape beforehand, thus facilitating the annotation process.

### Digital Breast Tomosynthesis Dataset ROIs Preprocessing

Each 2D ROI derived from the VOIs was used as an individual input. Before the training process, both ROIs and their respective binary masks were resized to 128 × 128 pixels, converted into 8-bit grayscale, and saved in TIFF format. No additional preprocessing steps were performed on the ROIs dataset. Our DBT ROIs dataset can be found under the name “BreasTomo-Synth” and has been made publicly available [54]. 

### Deep Learning Models

In this study, 13 models were created based on four well-established architectures for semantic segmentation: U-Net [40], Fully Convolutional Network (FCN) [56], DeepLabV3 [57], and DeepLabV3 + [58]. Firstly, U-Net is one of the most widely adopted segmentation architectures as it was designed for biomedical image segmentation with limited training data. Its U-shaped architecture with an encoder reduces spatial information while enhancing features, and a decoder that restores resolution through up-convolutions and feature concatenation.

In this study, U-Net is trained from scratch using its standard architecture. We defined this model as the baseline for further performance comparison analysis.

FCN, in turn, adapts a pretrained CNN for image classification into an encoder by replacing fully connected layers with convolutional layers, reusing their weights and biases. A decoder with a transposed convolution layer is added to upsample feature maps.

Finally, DeepLabV3 utilizes dilated convolutions to expand the receptive field and capture richer contextual information. Its Atrous Spatial Pyramid Pooling (ASPP) module captures multi-scale features. DeepLabV3 +, in turn, enhances the architecture by adding a decoder that upsamples feature maps and refines segmentation accuracy by combining them with low-level features.

We adopted ResNet-50 and ResNet-101 as backbones [59] since those types of convolutional neural network architectures are designed to mitigate the degradation problem in deep learning through residual connections, enabling efficient training of very deep models. With 50 and 101 layers respectively, these models are widely adopted for image recognition tasks and often serve as backbones in more complex network implementations. In the present study, we used the backbones pretrained on the COCO dataset [18], a large-scale benchmark for object detection, segmentation, and image captioning, featuring over 330,000 images and 1.5 million object instances across 80 categories. It emphasizes diverse, real-world scenes with rich contextual annotations.

### In Silico Models Comparative Analysis

We defined the U-Net as the baseline model. This architecture was compared with three other well-established segmentation architectures’ performances: FCN, DeepLabv3, and DeepLabv3 +. Two approaches were explored: (1) training from scratch using the in silico-generated dataset and (2) fine-tuning pretrained models with ResNet50 and ResNet101 as backbones. In total, 13 deep learning models were comparatively analyzed.

### In Silico Training Strategy and Code Implementation

An efficient region-of-interest-based (ROI-based) training strategy was implemented to optimize computational resources. By focusing on distinct ROI segments representing both surrounding healthy breast tissue and tumor regions, each treated as a unique sample, the method provides diverse examples for training. This approach enhances the deep learning network’s ability to generalize across various breast regions while minimizing computational costs.

Since the U-Net model takes input sizes that need to be divisible by 32, the 109 × 109 pixel 2D ROIs were resized to 128 × 128 pixels. No additional intensity normalization was applied. The UNet architecture training was only conducted from scratch using a binary cross-entropy (BCE) loss function, which is well-suited for binary segmentation tasks and helps optimize performance for pixel-wise classification.

The fine-tuning process for FCN, Deeplabv3, and Deeplabv3 + (with ResNet 50 or 101) was applied only to the final convolutional layers, while early layers remained frozen to retain feature extraction capabilities from the COCO dataset.

The Adam optimizer with a learning rate of 0.001 and a batch size of 20 was used for optimization. Additionally, stochastic weight averaging (SWA) was employed to average weights across multiple epochs, enhancing generalization and improving the model’s robustness. Batch normalization (BN) was employed to stabilize training and improve convergence by ensuring consistent feature distributions across mini-batches.

### In Silico Dataset Splitting

The dataset was divided using the stratified shuffle split method [60] to ensure that label distributions were preserved across all subsets. Initially, 80% of the data was allocated to training/validation, and 20% was reserved for testing. This stratification approach maintained consistent label proportions in all subsets, mitigating potential class imbalance.

Five re-shuffling splits were performed to support robust cross-validation, with a fixed random seed of 42, ensuring reproducibility.

During inference, the model produced pixel-wise probability maps via a sigmoid activation function. A threshold of 0.5 was applied to generate binary segmentation masks, classifying pixels as tumor or non-tumor. This threshold was chosen based on its ability to generate binary predictions that align well with the ground truth masks, thus optimizing performance metrics such as precision and recall. The code was implemented using PyTorch Lightning and ran using Google Colaboratory, with NVIDIA GPU T4 [61]. Table 1 summarizes the hyperparameter settings used in the code implementation for each segmentation model and both training approaches.

**Table 1:** Segmentation architectures and hyperparameters settings

Model	Loss function	Optimizer	Learning rate	Batch size	Max epochs	Training approach
U-Net	BCE	Adam	0.001	20	100	SCR
FCN	BCE	Adam	0.001	20	100	SCR, FT
Deeplabv3	BCE	Adam	0.001	20	100	SCR, FT
Deeplabv3 +	BCE	Adam	0.001	20	100	SCR, FT

*LR* learning rate, *BCE* binary cross entropy, *SCR* training from scratch, *FT* fine-tuned

### Optimization and Performance Metrics

As mentioned in the previous section, we utilized the Binary Cross Entropy (BCE) loss during the in silico models’ training. This optimization metric measures the discrepancy between the predicted and actual probability distributions for classification tasks. It is particularly suitable for pixel-level classification in segmentation [62].

The evaluation of semantic segmentation presents inherent complexity, as it requires assessing both classification accuracy and localization precision. The objective is to quantify the degree of similarity between the predicted segmentation output and the annotated reference standard (ground truth). Thus, the performance metrics selected for our study were F1-score, precision, recall, and intersection over union (IoU) [63]. These metrics collectively provide a robust framework for evaluating and optimizing the performance of segmentation models, balancing accuracy and precision in diverse scenarios.

### Statistical Analysis

A pairwise statistical analysis was conducted to compare the performance of U-Net from scratch, which was used as the baseline model, with the other 12 deep learning models’ variants, either trained from scratch or pretrained and fine-tuned using ResNet 50 or 101 as backbones. Given that we assumed the non-parametric nature of the data, the Wilcoxon Signed-Rank Test was applied to the performance metrics (F1-score, Intersection over Union (IoU), Precision, and Recall). The Bonferroni correction was applied to account for multiple comparisons, setting the adjusted significance threshold at *α* = 0.0042 (calculated as *α* = 0.05/12, where 12 is the number of pairwise comparisons).

### Hybrid Dataset and Training Strategy

To evaluate generalization across domains, we designed a hybrid training experiment that combined in silico-generated ROIs with real-world DBT ROIs. We leveraged 230 simulated in silico ROIs from our previous experiment and then extracted a subset of real-world tumor ROIs from the publicly available Breast Cancer Screening-Digital Breast Tomosynthesis (BCS-DBT) (version 5) dataset by Buda et al. [13], hosted at The Cancer Imaging Archive [64]. From the 38 malignant lesions belonging to the BCS-DBT dataset volumes we extracted the central ROIs from real tumor cases and combined the data with the already existing in silico dataset. We excluded cases containing either architectural distortions (AD) or tumoral masses with metallic clip markers in the surrounding tissue. Thus, a total of 20 central slice tumor ROIs were integrated into the new dataset of 250 hybrid DBT ROIs. Images preprocessing, resizing, and masks delineation procedures were the same as described in the previous section for in silico ROIs.

The 250 hybrid DBT ROIs dataset was used to retrain the baseline U-Net model, and its performance was assessed on a separate holdout set to determine the effectiveness of combining both data sources for tumor segmentation tasks. This method consists of dividing the 250 hybrid ROIs into 200 for training (80%), 25 for validation, and 25 for testing. Training is performed using 200 samples, with validation conducted using another 25 validation samples between epochs. Once training is completed, performance metrics are evaluated using the 25 hybrid test samples.

Finally, after the hybrid model was trained and evaluated with the hybrid approach, predictions were made using a new, independent subset composed of 20 real-world data ROI samples derived from the BSC-DBT dataset.

The Dice loss function has been implemented in this hybrid dataset experiment, as it is a widely used metric and loss function for biomedical image segmentation due to its robustness to class imbalance [65]. All the remaining hyperparameter settings are similar to the in silico experiment.

## Results

### Training and Validation Loss Curve Dynamics for the Deep Learning Models

**Fig. 4:**
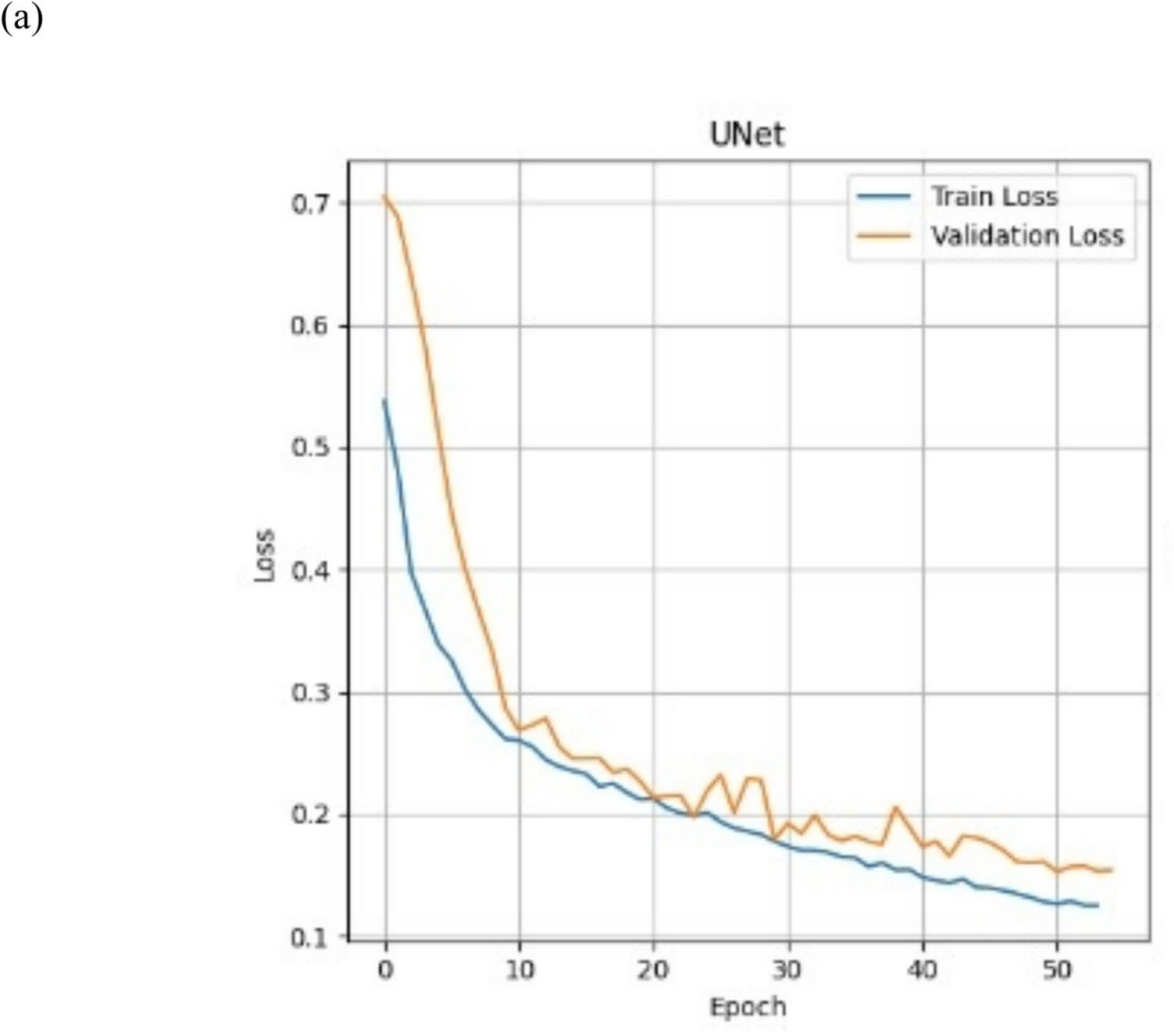

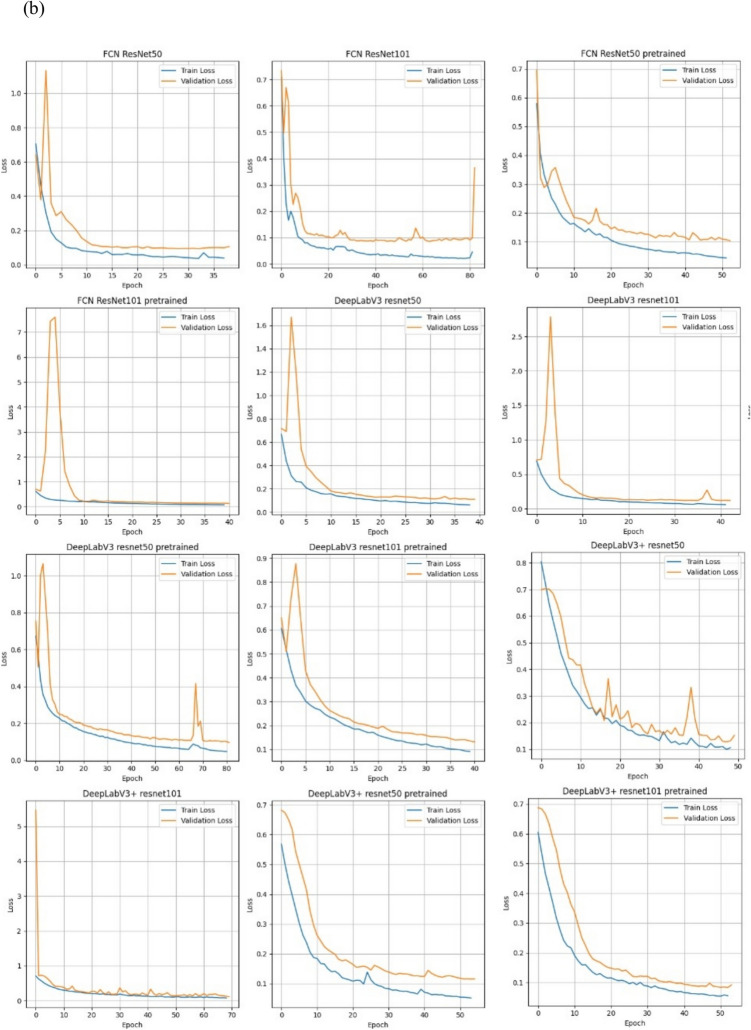
**a** Training and validation BCE loss over epochs for U-Net trained from scratch on the in silico DBT dataset. The model shows smooth convergence and minimal divergence between training and validation curves, indicating stable training and low overfitting risk. **b** Training and validation BCE loss curves for the remaining 12 deep learning models: FCN, DeepLabv3, and DeepLabv3 + architectures with ResNet50 and ResNet101 backbones, trained either from scratch or fine-tuned on COCO-pre-trained weights. Most models converge stably, though several pretrained configurations exhibit fluctuations in validation loss, suggesting variability in generalization likely due to domain shift from natural to in silico images

To assess the training dynamics and convergence behavior of each deep learning model, we analyzed the binary cross-entropy (BCE) loss over training epochs for all 13 model configurations. These included models trained from scratch and those fine-tuned from COCO-pretrained weights across four architectures: U-Net, FCN, DeepLabv3, and DeepLabv3 +. In general, most models exhibited a consistent downward trend in training loss, with validation loss closely tracking training performance. U-Net trained from scratch displayed stable convergence with minimal overfitting, while some pretrained models showed higher variance in validation loss. This dynamic behavior suggests challenges in domain adaptation from natural to medical image distributions. Figure 4a shows the baseline U-Net from scratch loss curve dynamics, and Fig. 4b show the remaining 12 models’ loss curve dynamics.

### Deep Learning Models Performance Metrics Comparative Analysis

Table 2 presents the comparative performance of 13 deep learning models evaluated on the in silico DBT dataset using F1-score, Intersection over Union (IoU), Precision, and Recall. U-Net trained from scratch achieved the highest overall performance among all models, with an F1-score of 82.52%, IoU of 78.49%, precision of 90.30%, and recall of 75.97%, demonstrating strong segmentation capability and balanced precision-recall characteristics. Among the remaining models, DeepLabv3 + with ResNet50 fine-tuned on COCO (Pretrained FT) achieved the best performance, with an F1-score of 84.98% and the highest IoU of 83.77%, indicating that fine-tuning in certain architectures can improve spatial overlap without compromising precision (94.45%) or recall (77.23%).

**Table 2:** Quantitative comparison of deep learning models for tumor segmentation on in silico DBT data using F1-score, IoU, precision, and recall

Model	F1-score (%)	IoU (p/img) (%)	Precision (%)	Recall (%)
U-NET	**82.52**	78.49	90.3	**75.97**
FCN RN50 SCR*	76.88	78.47	93.26	65.39
FCN RN101 SCR	46.36	57.96	83.03	32.16
FCN RN50 Pretrained FT**	77.46	82.8	93.22	66.25
FCN RN101 Pretrained FT	72.97	79.68	87.81	62.42
DeepLabv3 RN50 SCR	78.22	78.85	92.05	68.01
DeepLabv3 RN101 SCR	77.55	79.12	88.9	68.77
DeepLabv3 RN50 Pretrained FT	77.83	80.83	92.12	67.38
DeepLabv3 RN101 Pretrained FT	76.54	77.73	90.06	66.34
DeepLabv3 + RN50 SCR	72.25	67.69	77.34	67.79
DeepLabv3 + RN101 SCR	73.29	72.27	92.17	60.84
DeepLabv3 + RN50 Pretrained FT	**84.98**	**83.77**	**94.45**	**77.23**
DeepLabv3 + RN101 Pretrained FT	81.03	77.0	90.45	73.53

*RN50* ResNet 50 backbone, *RN100* ResNet 100 backbone, *SCR scratch, **FT fine-tuned, *p/img* per image

However, performance varied widely across models, especially within the FCN family. Notably, FCN101 trained from scratch showed poor generalization, with the lowest F1-score (46.36%) and recall (32.16%), likely reflecting its reduced capacity to learn meaningful features from the in silico data. Fine-tuned FCN and DeepLabv3 variants showed moderate results but still exhibited signs of instability in recall performance. In general, models pretrained on natural image datasets did not consistently outperform their scratch-trained counterparts, emphasizing the potential limitations of transfer learning in domain-shifted contexts. These results support the notion that architecture choice and training strategy must be carefully matched to the data domain, particularly when leveraging in silico medical imaging data.

### Visual Comparative Analysis of In Silico Deep Learning Models

**Fig. 5:**
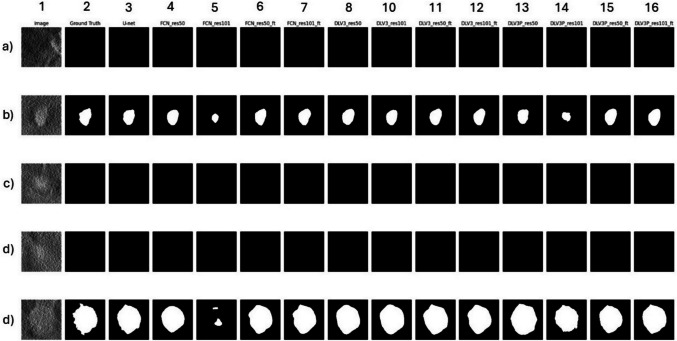
Segmentation results for five test cases (rows a–d) from the in silico DBT dataset. Columns show the original image (1), ground truth (2), and predictions from 13 deep learning models (columns 3–16), including U-Net, FCN, DeepLabv3, and DeepLabv3 + architectures trained from scratch or fine-tuned with ResNet50/101. U-Net (column 3) and DeepLabv3 + ResNet50 fine-tuned (column 13) produced the most accurate and consistent masks. FCN models (columns 4–7) failed to detect lesions in most cases, while DeepLabv3 variants (columns 8–12) showed moderate performance

Figure 5 displays a side-by-side qualitative comparison of segmentation outputs across five representative test cases (Rows a–d) from the in silico DBT dataset. Each row corresponds to a different test image, while the columns show the original DBT slice (column 1), ground truth mask (column 2), and predictions from 13 deep learning models (Columns 3–16). These include U-Net, FCN, DeepLabv3, and DeepLabv3 + architectures, trained from scratch or fine-tuned with COCO-pretrained ResNet50 and ResNet101 backbones.

The U-Net model (column 3) consistently produced segmentation masks that closely matched the ground truth, particularly evident in Rows b and d. DeepLabv3 + fine-tuned with ResNet50 (column 15) also demonstrated high-quality predictions, showing accurate tumor boundaries and minimal false positives. In contrast, FCN-based models (columns 4–7) failed to detect any lesions across most cases, including both scratch-trained and fine-tuned variants. DeepLabv3 models (columns 8–12) showed moderate performance, occasionally capturing tumor regions but with reduced spatial precision or under-segmentation, especially in rows b and d. Overall, this visual analysis supports the quantitative findings: U-Net and DeepLabv3 + (ResNet50 FT) were the most effective at generalizing from in silico data. The observed discrepancies across models highlight the influence of architecture, training paradigm, and feature representation capacity when deploying segmentation models on synthetic breast imaging data.

### Statistical Comparative Analysis of Deep Learning Models

This section reports the statistical analysis comparing the performance of deep learning models for in silico breast tumor segmentation. Based on the comparative performance results presented in Table 2, U-Net trained from scratch achieved the highest overall performance across all evaluation metrics, including F1-score (82.52%), IoU (78.49%), Precision (90.30%), and Recall (75.97%). To assess the statistical significance of these differences, U-Net was used as the reference model in pairwise comparisons against all other architectures, encompassing both scratch-trained and fine-tuned variants of FCN, DeepLabv3, and DeepLabv3 + with ResNet50 and ResNet101 backbones. The Wilcoxon signed-rank test, followed by Bonferroni correction for multiple comparisons, was applied to evaluate differences across F1-score, IoU, Precision, and Recall. The results, summarized in Table 3 (Appendix), showed no statistically significant differences (adjusted *p* > 0.0042) between U-Net and any of the other models. While U-Net consistently led in performance, particularly when compared to FCN variants and several pretrained models, these differences were not statistically significant under the evaluation criteria, suggesting performance comparability among the top models in this experimental setting.

### Hybrid Training Dice Loss Curve Dynamics for U-Net

**Fig. 6:**
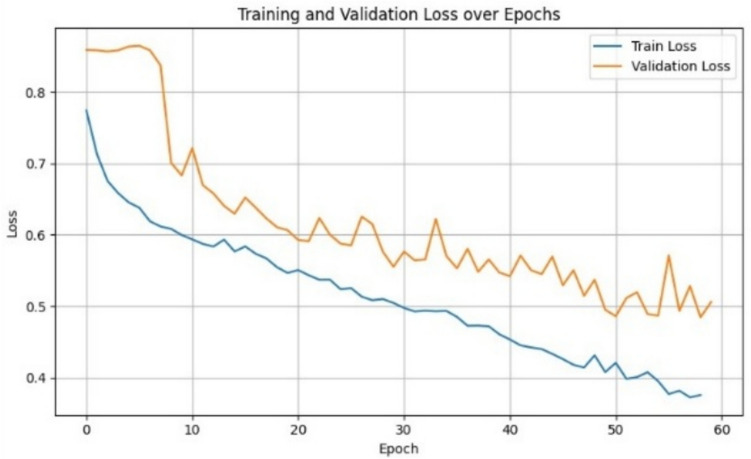
Training and validation Dice loss curves for the U-Net model trained on the hybrid dataset. The loss steadily decreases across 60 epochs, indicating stable convergence and moderate signs of overfitting. The use of Dice loss is particularly suited for class-imbalanced segmentation tasks, such as tumor delineation in DBT

The baseline U-Net model was retrained using the 250-ROI hybrid DBT dataset as described in the methods section, comprising both in silico and real-world examples, with an 80/10/10 split for training, validation, and testing. The Dice loss function was used to optimize segmentation performance, particularly due to its robustness in handling class imbalance, a common issue in tumor segmentation. As shown in Fig. 6, the Dice loss curve demonstrates a consistent downward trend across 80 epochs, with training and validation losses closely aligned and steadily decreasing. This suggests stable convergence and minimal overfitting during model training.

**Fig. 7:**
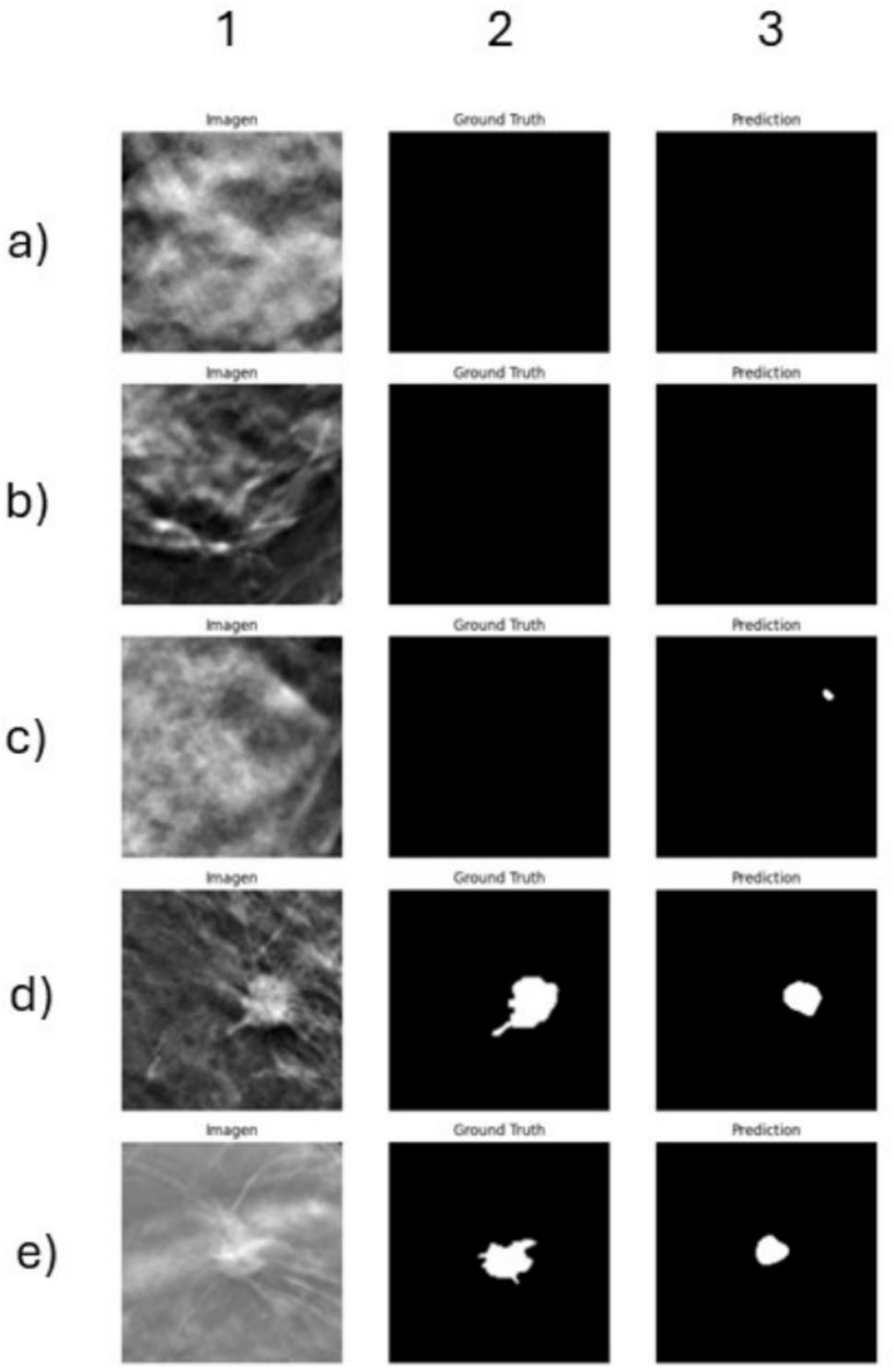
Qualitative segmentation results of the U-Net model trained on the hybrid DBT dataset and evaluated on 20 real-world DBT independent test samples. Each row (a–e) corresponds to a different test image ROI. Columns represent (1) the original DBT central slice ROI, (2) the ground truth binary mask, and (3) the model prediction. Rows a–c correspond to healthy breast tissue with an empty ground truth mask, which were correctly predicted as negative cases. A small false-positive region is observed in row c, column 3. Rows d and e depict Tumor-containing ROIs, with the predicted masks showing good overlap with the ground truth, capturing the core lesion despite slight under-segmentation. These results demonstrate the model’s ability to generalize to real-world data in both negative and positive cases, while highlighting remaining challenges in detecting subtle or low-contrast lesions

Quantitative results on the test set indicate strong segmentation performance: the model achieved an F1-score of 79%, per-image IoU of 76%, and dataset level IoU of 65%, with a notably high precision of 90% and a recall of 70%. These metrics reflect the model’s capacity to identify tumor regions accurately while maintaining a low false-positive rate. Visual inspection of representative real-world test cases (Fig. 7) shows that the model correctly delineates tumors in high-contrast scenarios, while under-segmentation is observed in more complex or low-contrast cases. Overall, these results indicate that hybrid training with in silico and limited real-world data may support model generalization to some extent, although the influence of domain shift and dataset diversity warrants further investigation.

## Discussion

This study evaluated the viability of using an in silico-generated Digital Breast Tomosynthesis (DBT) dataset as an alternative data source by assessing the performance of various well-established deep learning models for breast tumor segmentation. Our findings demonstrated that U-Net trained from scratch and DeepLabv3 + with ResNet50 pretrained on COCO achieved the highest performance among all configurations, with F1-scores of 82.52% and 84.98%, and per-image IoUs of 78.49% and 83.77%, respectively. Pretrained models showed competitive precision but exhibited more variability in recall and convergence dynamics, likely due to domain shift from natural image datasets. These findings align with recent literature indicating that while pretrained architectures may accelerate convergence in some tasks, training from scratch on domain-specific datasets remains preferable in specialized biomedical imaging contexts [65–67].

Our in silico dataset, generated using the FDA-cleared VICTRE software, incorporated a wide range of breast densities and tumor complexities, providing a rich training ground despite its relatively limited size (n = 230). The similar simulated images with the VICTRE software have been validated in previous work against real images distributions in terms of contrast-to-noise ratio and texture features [33, 38, 45].

While training was conducted on 2D ROIs, extracting them from anatomically realistic full-volume simulations allowed us to preserve tissue complexity and imaging fidelity, offering a practical compromise between clinical realism and computational efficiency.

Additionally, using a stratified shuffle-split ensured balanced class representation and tumor diversity across subsets, minimizing the risk of overfitting. This training strategy, paired with the use of Stochastic Weight Averaging and Batch Normalization, resulted in stable training behavior as evidenced by the loss dynamics.

A visual inspection of predictions confirmed these trends: U-Net and DeepLabv3 + (with ResNet50 fine-tuned) provided accurate tumor segmentation with fewer false positives, while FCN variants underperformed across test cases. Notably, statistical analysis showed no significant differences between models (Wilcoxon test with Bonferroni correction, *α* > 0.0042), reinforcing that in this experimental setting, multiple architectures offer similar baseline utility when trained on high-quality in silico data.

A major contribution of this work is the inclusion of a hybrid training experiment to explore generalization capacity. The U-Net model was retrained on a dataset of 250 hybrid ROIs, including both in silico and real-world DBT ROIs. A new, independent subset composed of 20 real-world ROIs was used for predictions.

Despite the limited number of real-world cases, the hybrid model achieved promising results, with an F1-score of 79%, per-image IoU of 76.4%, dataset-level IoU of 65.4%, precision of 90.8%, and recall of 70%. These outcomes suggest that combining real and synthetic data may improve generalizability and mitigate the limitations imposed by small real-world clinical datasets, even in the presence of domain shift. However, further research using a larger real-world dataset is needed to validate and extend these preliminary findings.

Our hybrid training approach also demonstrated the capacity to segment real-world DBT samples, correctly identifying healthy and tumorous ROIs. While segmentation accuracy declined in complex or low-contrast cases, the results represent a foundational step toward bridging the gap between synthetic and real-world clinical data in training AI models. The use of Dice loss in this second experiment further improved robustness to class imbalance, an important factor encountered in medical imaging tasks.

Nonetheless, limitations must be acknowledged. The real-world data sample was small and limited to ROIs extracted from only one public DBT dataset. Additionally, our methodology was based on 2D slices rather than full 3D volumes, which limits contextual information. As such, results should be interpreted conservatively as part of an exploratory investigation into hybrid training strategies for DBT segmentation.

Future work should focus on expanding the real-world DBT dataset for improved generalization, exploring 3D volumetric segmentation, and integrating domain adaptation methods such as histogram matching or adversarial learning to further align distributions between synthetic and real clinical data. Moreover, investigating other generative modeling strategies (e.g., diffusion models or GANs) may enhance realism and diversity in synthetic data creation.

## Conclusion

This exploratory study aimed to evaluate the viability of in silico-generated DBT data as a complementary training resource for breast tumor segmentation using deep learning. U-Net and DeepLabv3 + demonstrated the most consistent performance when trained on our in silico dataset, with no significant statistical difference found between models. The high-quality in silico dataset, built using an FDA-cleared simulation pipeline, enabled stable training and generalizable model performance, particularly when paired with robust partitioning and training techniques.

To enhance generalization, we retrained the U-Net model using a hybrid dataset combining in silico and real-world ROIs. Despite the modest size of the clinical subset, the model maintained promising segmentation accuracy on independent real-world test samples. These findings suggest that in silico datasets can serve as a useful supplement in scenarios with limited annotated clinical data, although further validation is needed.

While our results are encouraging, they should be interpreted as a proof-of-concept. The integration of synthetic and real-world clinical data requires continued refinement to ensure reliability and applicability in real-world clinical workflows. Future directions include scaling up clinical validation, extending to 3D segmentation, and adopting domain adaptation techniques to enhance cross-domain robustness.

## Figures and Tables

**Fig. 8:**
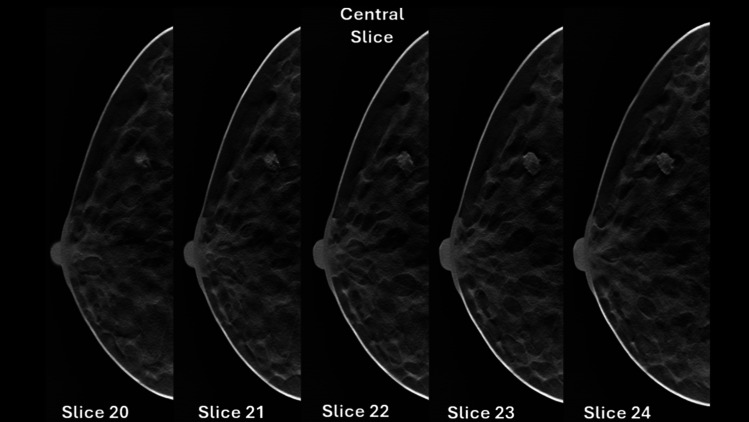
Example of a DBT reconstruction with a dimension of 1181 × 480 × 52 (height, width, and depth, respectively) derived from a virtual breast phantom simulation at a 70% fat fraction from the BI-RADS © scattered density category. A spiculated tumor with a mean radius of 2.30 mm is inserted in slice 22 (the tumor’s central slice) and spans several slices. For illustration purposes only; slices 20 to 24 are represented here

## Data Availability

The original data presented in the study are openly available in Zenodo at https://doi.org/10.5281/zenodo.14270329. Digital Breast Tomosynthesis (BCS-DBT) (Version 5) is available in The Cancer Imaging Archive at https://doi.org/10.7937/E4WT-CD02

## Appendix

**Table 3:** Wilcoxon signed-rank test, with a post hoc Bonferroni correction for multiple pairwise comparisons between U-Net and the other 12 deep learning models

Model	F1-score			IoU			Precision			Recall		
	Raw	C	S	Raw	C	S	Raw	C	S	Raw	C	S
FCN R50 SCR	0.5	1.0	No	0.5	1.0	No	0.5	1.0	No	0.5	1.0	No
FCN R101 SCR	0.5	1.0	No	0.5	1.0	No	0.5	1.0	No	0.5	1.0	No
FCN R50 FT	0.5	1.0	No	0.5	1.0	No	0.5	1.0	No	0.5	1.0	No
FCN 101 FT	0.5	1.0	No	0.5	1.0	No	0.5	1.0	No	0.5	1.0	No
DLV3 R50 SCR	0.5	1.0	No	0.5	1.0	No	0.5	1.0	No	0.5	1.0	No
DLV3 R101 SCR	0.5	1.0	No	0.5	1.0	No	0.5	1.0	No	0.5	1.0	No
DLV3 R50 FT	0.5	1.0	No	0.5	1.0	No	0.5	1.0	No	0.5	1.0	No
DLV3 R101 FT	0.5	1.0	No	0.5	1.0	No	0.5	1.0	No	0.5	1.0	No
DLV3 + R50 SCR	0.5	1.0	No	0.5	1.0	No	0.5	1.0	No	0.5	1.0	No
DLV3 + R101 SCR	0.5	1.0	No	0.5	1.0	No	0.5	1.0	No	0.5	1.0	No
DLV3 + R50 FT	0.5	1.0	No	0.5	1.0	No	0.5	1.0	No	0.5	1.0	No
DLV3 + R101 FT	0.5	1.0	No	0.5	1.0	No	0.5	1.0	No	0.5	1.0	No

*R50* ResNet 50 backbone, *R101* ResNet 101 backbone, *SCR* scratch, *FT* fine-tuned, *C* corrected, *S* significance

## Code Availability

The code is available at https://github.com/nickarcaro/insilicoDBT
